# Supplementary material for: Numb positively regulates Hedgehog signaling at the ciliary pocket
Source: Nat Commun. 2024 Apr 25;15:3365. doi: 10.1038/s41467-024-47244-1 (PMC11045789; doi:10.1038/s41467-024-47244-1)
Supplement: Supplementary file 1 — Supplementary Information [file 41467_2024_47244_MOESM1_ESM.pdf]

Supplementary information for

Numb positively regulates Hedgehog signaling at the ciliary pocket

Xiaoliang Liu[1,9], Patricia T. Yam[2,9], Sabrina Schlienger[2,6], Eva Cai[1], Jingyi Zhang[1], Wei-Ju Chen[2,5], Oscar Torres Gutierrez[1], Vanesa Jimenez Amilburu[2], Vasanth Ramamurthy[2], Alice Y. Ting[3], Tess C. Branon[3,4], Michel Cayouette[2,6,7], Risako Gen[8], Tessa Marks[8], Jennifer H. Kong[8], Frédéric Charron[2,6,7*], Xuecai Ge[1*]

[1]Department of Molecular and Cell Biology, University of California, Merced, Merced California, 95340, USA.
[2]Montreal Clinical Research Institute (IRCM), Montreal, QC H2W 1R7, Canada.
[3]Departments of Genetics, of Biology, and by courtesy, of Chemistry, Stanford University, Stanford, California, USA. Chan Zuckerberg Biohub, San Francisco, California, USA.
[4]Current address: Interline Therapeutics, South San Francisco, CA, USA.
[5]Department of Biology, McGill University, Montreal QC H3A 0G4, Canada.
[6]Department of Anatomy and Cell Biology, McGill University, Montreal QC H3A 0G4, Canada.
[7]Department of Medicine, University of Montreal, Montreal QC H3T 1J4, Canada.
[8]Department of Biochemistry, University of Washington, Seattle, WA, 98195
[9]These authors contributed equally.

*Correspondence: frederic.charron@ircm.qc.ca (F.C.), xge2@ucmerced.edu (X.G.)

The PDF file includes:

Supplementary Figures and figure legends

Supplementary Figure 1: Truncated Arl13b localizes to the cilium with minimal impact on cilium length in transiently transfected cells.
Supplementary Figure 2: Cilium-TurboID in stable cell lines does not impact cilium morphology and Hh signal transduction.
Supplementary Figure 3: Analysis of mass spectrometry results.
Supplementary Figure 4: Correlation of biological replicates in mass spectrometry.
Supplementary Figure 5: Numb binds to Ptch1 via Numb-N and PTB domain.
Supplementary Figure 6: YFP control does not show any co-localization with Numb.
Supplementary Figure 7: Design of *Numb* CRISPR/Cas9 and identification of genomic mutations in individual cell clones.
Supplementary Figure 8: Numb is required for Shh-induced Ptch1-YFP exit from the cilium.
Supplementary Figure 9: *Numb* shRNA impairs activation of Hh signaling.
Supplementary Figure 10: Activating signaling components downstream of Ptch1 turns on Hh signaling in Numb KO cells.
Supplementary Figure 11: The effect of Numb loss on ciliary accumulation of Smo.
Supplementary Figure 12: *Numblike* knockdown does not impact Hh signaling.
Supplementary Figure 13: Numb loss has moderate or no impact on the differentiation of NPCs that is reliant on medium to low Hh signaling activity.
Supplementary Figure 14: Numb is required for activation of Hh signaling and Shh-induced Ptch1 exit from the cilium in GCPs.
Supplementary Figure 15: *Numb/Numbl* cDKO leads to reduced cerebellar size in adult mice.

Supplementary Methods

Chemicals and other reagents used in this study
Quantitative real-time PCR

Supplementary References

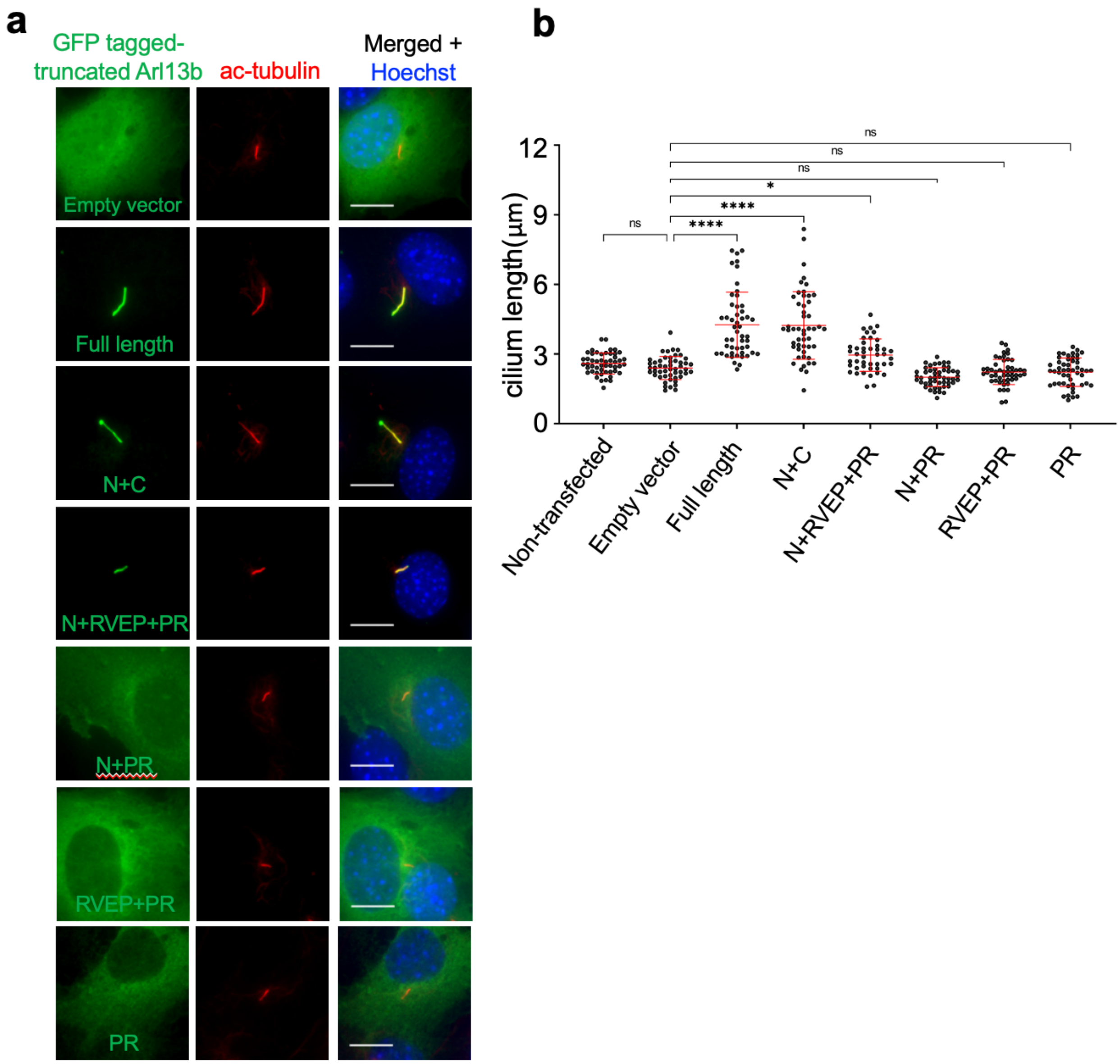

Supplementary Figure 1. Truncated Arl13b localizes to the cilium with minimal impact on cilium length in transiently transfected cells.

(**a**) GFP-tagged truncated Arl13b constructs were transiently expressed in NIH3T3 cells. Cells were immunostained with an antibody against GFP and the cilium marker acetylated-tubulin. Scale bar, 10 µm. (**b**) Quantification of the cilium length in cells transiently transfected with truncated Arl13b constructs. Note that in the stable cell lines in supplementary Figure 2A where the expression levels are much lower, N+RVEP+PR has no significant impact on cilium length. Data are shown as mean ± SD. A total of 50 cilia were quantified per condition. One-way ANOVA with multiple comparisons (Tukey test). ****, $p < 0.0001$; *, $p < 0.05$; ns, not significant. Scale bars, 10 µm in all panels. Source data are provided as a Source Data file.

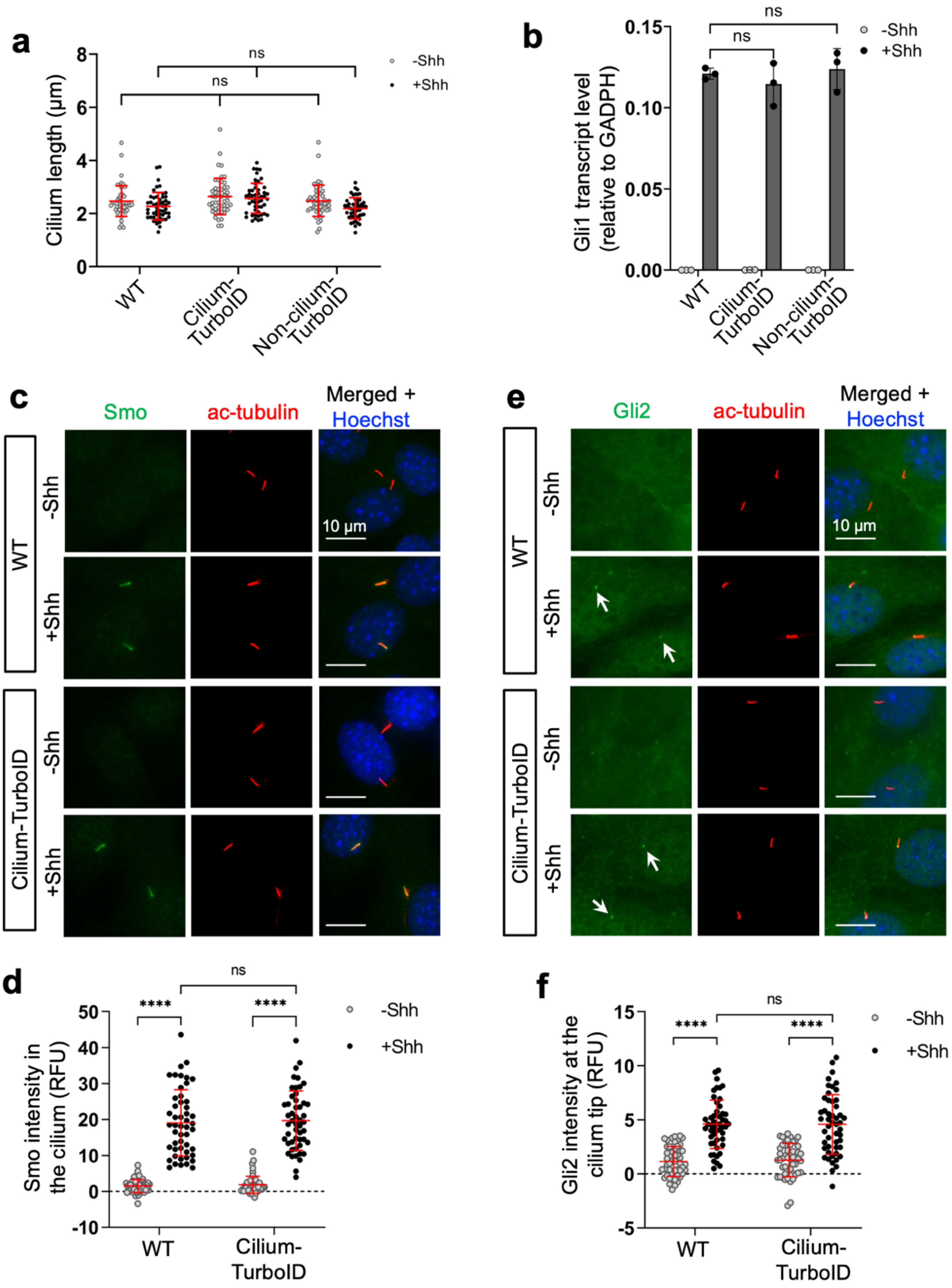

Supplementary Figure 2. Cilium-TurboID in stable cell lines does not impact cilium morphology and Hh signal transduction.

(**a**) Quantification of the cilium length in WT (parental NIH3T3 cells), Cilium-TurboID and Non-cilium-TurboID stable cell lines. A total of 50 cilia were quantified per condition.
(**b**) Cells were stimulated with Shh for 24h, and Hh signaling activity was evaluated by qPCR measuring *Gli1* transcript levels. Data are shown as relative transcript levels normalized to *GAPDH* (n = 3). (**c**, **e**) Immunofluorescence of Smo and Gli2 in WT and Cilium-TurboID stable cell lines. Cells were serum-starved with or without Shh in the culture medium for 24 h. Arrows in (**d**) point to Gli2 fluorescence signal at cilia. (**d**, **f**) Quantification of Smo and Gli2 fluorescence intensity in the cilium. Data are shown as mean ± SD. A total of 50 cilia were quantified per condition. RFU, relative fluorescence unit. Statistics in (**a**, **b**, **d** and **f**): Two-way ANOVA with multiple comparisons (Tukey test), ****, $p < 0.0001$. ns, not significant. Scale bars, 10 μm in all panels. Source data are provided as a Source Data file.

a The distribution of samples into TMT channels

Label in Fig2c	TMT channel	Experimental condition
A1	126	CiliumTurboID: +biotin +shh
B1	127C	CiliumTurboID: +biotin -shh
C1	127N	CiliumTurboID: -biotin +shh
D1	128N	nonciliumTurboID: +biotin +shh
A2	128C	CiliumTurboID: +biotin +shh
B2	129C	CiliumTurboID: +biotin -shh
C2	129N	CiliumTurboID: -biotin +shh
D2	130N	nonciliumTurboID: +biotin +shh
A3	130C	CiliumTurboID: +biotin +shh
B3	131C	CiliumTurboID: +biotin -shh
C3	131N	CiliumTurboID: -biotin +shh
D3	132N	nonciliumTurboID: +biotin +shh
	134N	Pooled

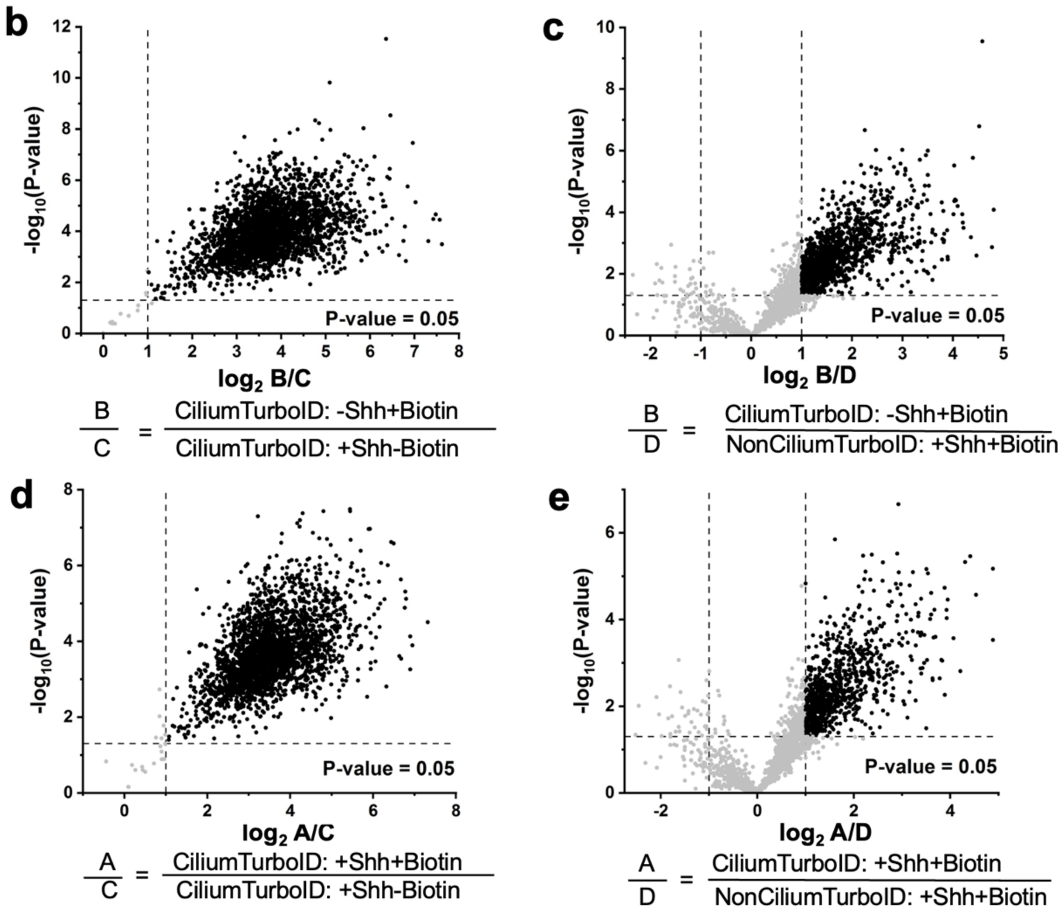

Supplementary Figure 3. Analysis of mass spectrometry results.
(**a**) The distribution of samples in the channels of TMTpro 16plex label reagent set.
(**b-e**) Analysis was performed on 2,880 proteins that have more than 7 unique peptides. Volcano plots were generated by plotting the $\log_2$ TMT ratio of the indicated conditions against the negative $\log_{10}$ p-value of Student's t-test of the corresponding conditions. The P values were adjusted for

multiple testing via the Benjamini-Hochberg procedure. In each plot, dashed lines indicate the cutoffs of $p < 0.05$ and TMT ratio > 2 or <-2. Proteins that meet the cutoffs are represented by black dots. The proteins that meet the cutoffs were further analyzed as follows.

(1) The intersection of proteins in (**b**) (2643 candidates) and in (**c**) (1225 candidates) are defined as ciliary proteins without Shh stimulation; 788 proteins are identified in this category (Supplementary Data 1).

(2) The intersection of proteins in (**d**) (2636 candidates) and in (**e**) (890 candidates) are defined as ciliary proteins with Shh stimulation; 574 proteins are identified in this category (Supplementary Data 1).

(3) The union of candidates identified in step (1) and (2) are defined as ciliary candidates. 800 proteins are identified in this category (Supplementary Data 2). These 800 proteins are plotted in Fig. 2c and Fig. 2e. Source data are provided as a Source Data file.

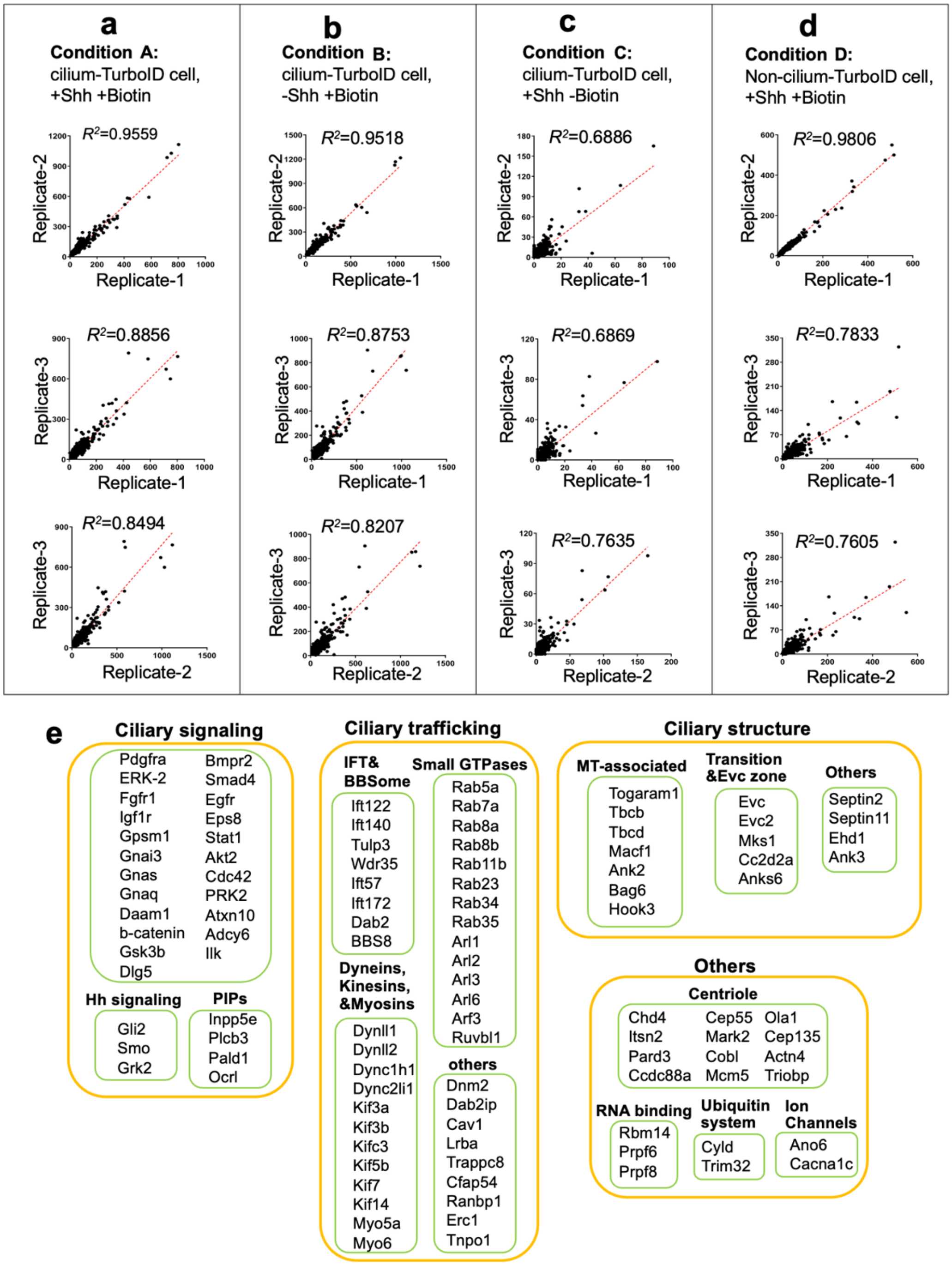

Supplementary Figure 4. Correlation of biological replicates in mass spectrometry.
(**a**) Correlation of biological replicates for condition a: cilium-TurboID cells labeled with biotin and stimulated with Shh. The averaged R-squared is 0.8969. (**b**) Correlation of biological replicates for condition b: cilium-TurboID cells labeled with biotin without Shh. The averaged R-squared is 0.8826. (**c**) Correlation of biological replicates for condition c: cilium-TurboID cells stimulated with Shh without biotin labeling. The averaged R-squared is 0.7130. (**d**) Correlation of biological replicates for condition d: non-cilium-TurboID cells labeled with biotin and stimulated with Shh. The averaged R-squared is 0.8415. (**e**) The previously reported ciliary candidates recovered in this study are listed and grouped into functional categories.

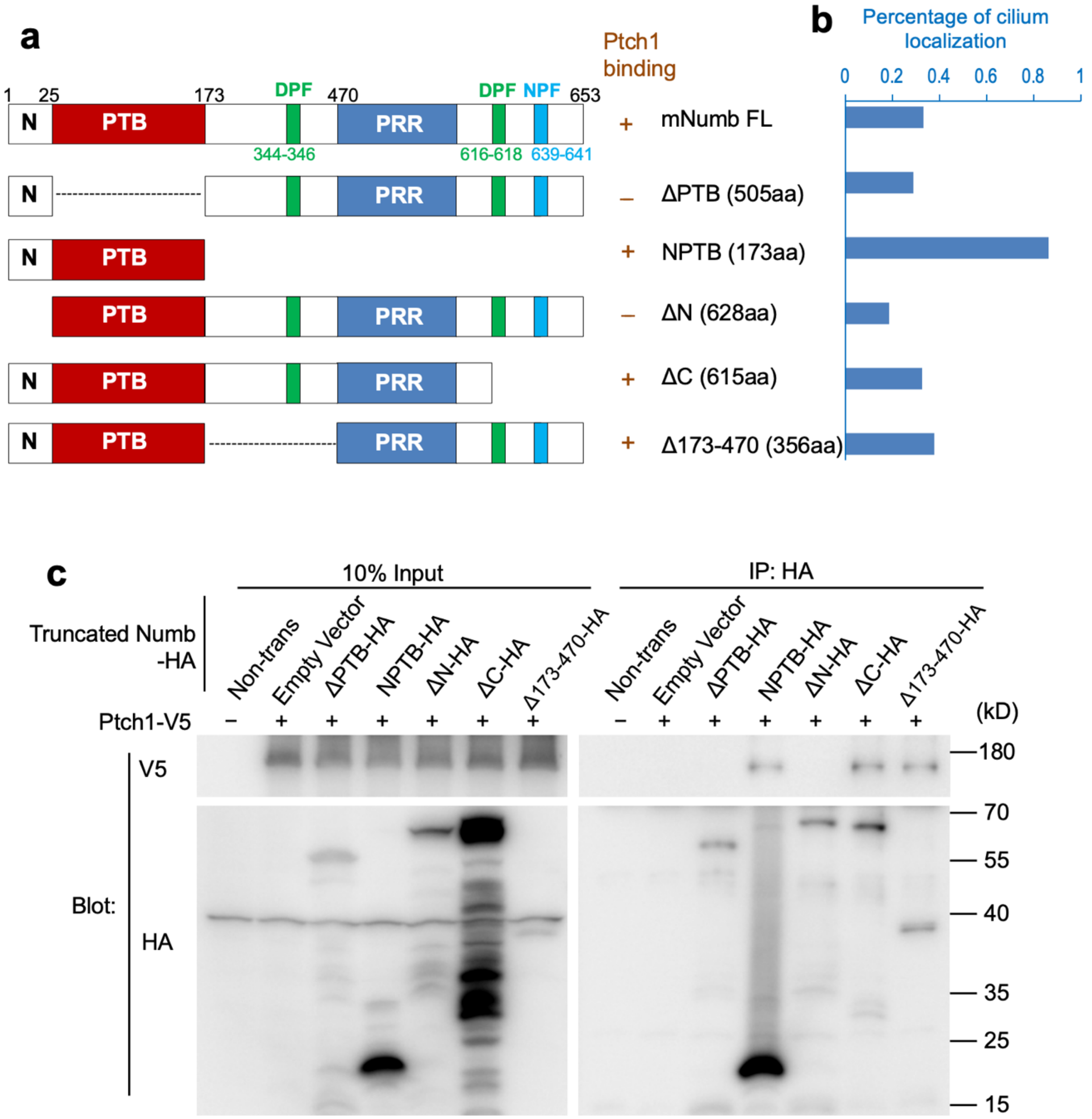

Supplementary Figure 5. Numb binds to Ptch1 via Numb-N and PTB domain.
(**a**) Schematic representation of Numb protein structure and truncated Numb variants used to map the interaction domain with Ptch1. (**b**) Truncated Numb variants were expressed in NIH3T3 cells via lentivirus, and the percentage of cilium localization for all variants were quantified in transfected cells. (**c**) Ptch1-V5 and HA tagged-Numb-truncated proteins were expressed in 293T cells. Co-immunoprecipitation was done with an anti-HA antibody. Deletion of either the N or PTB domain disrupts the interaction between Numb and Ptch1, while the N+PTB domain itself is sufficient to mediate the binding with Ptch1-V5. Source data are provided as a Source Data file.

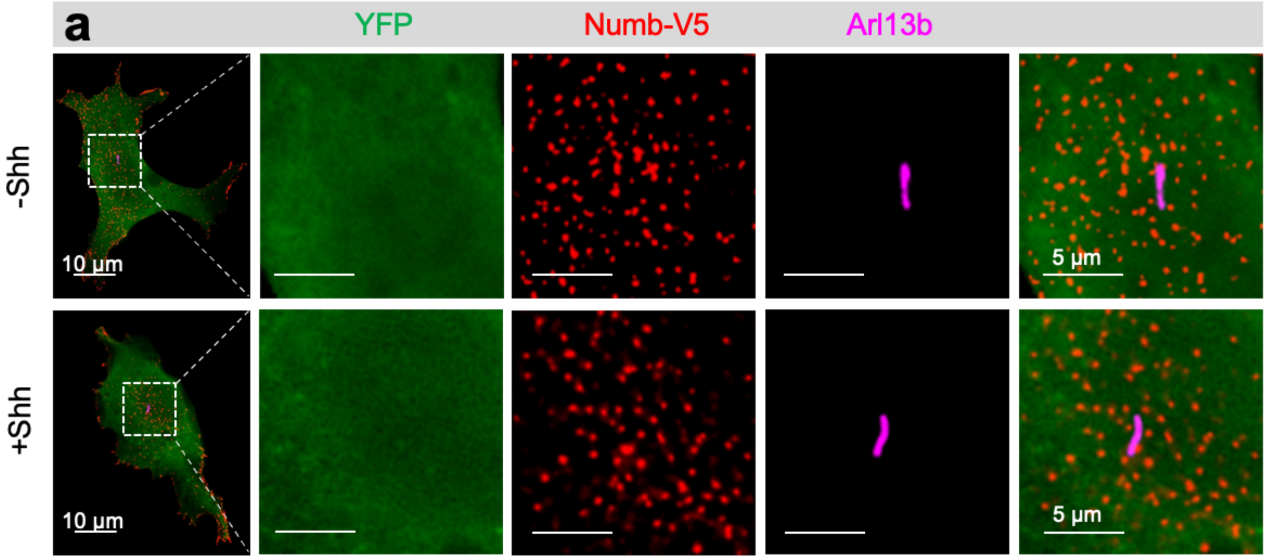

Supplementary Figure 6. YFP control does not show any co-localization with Numb.
(**a**) As a control for Figure 3h, YFP protein was expressed in NIH3T3 cells, together with Numb-V5. Cells were treated with Shh or vehicle for 30 min before fixation and staining. YFP control does not show any co-localization with Numb.

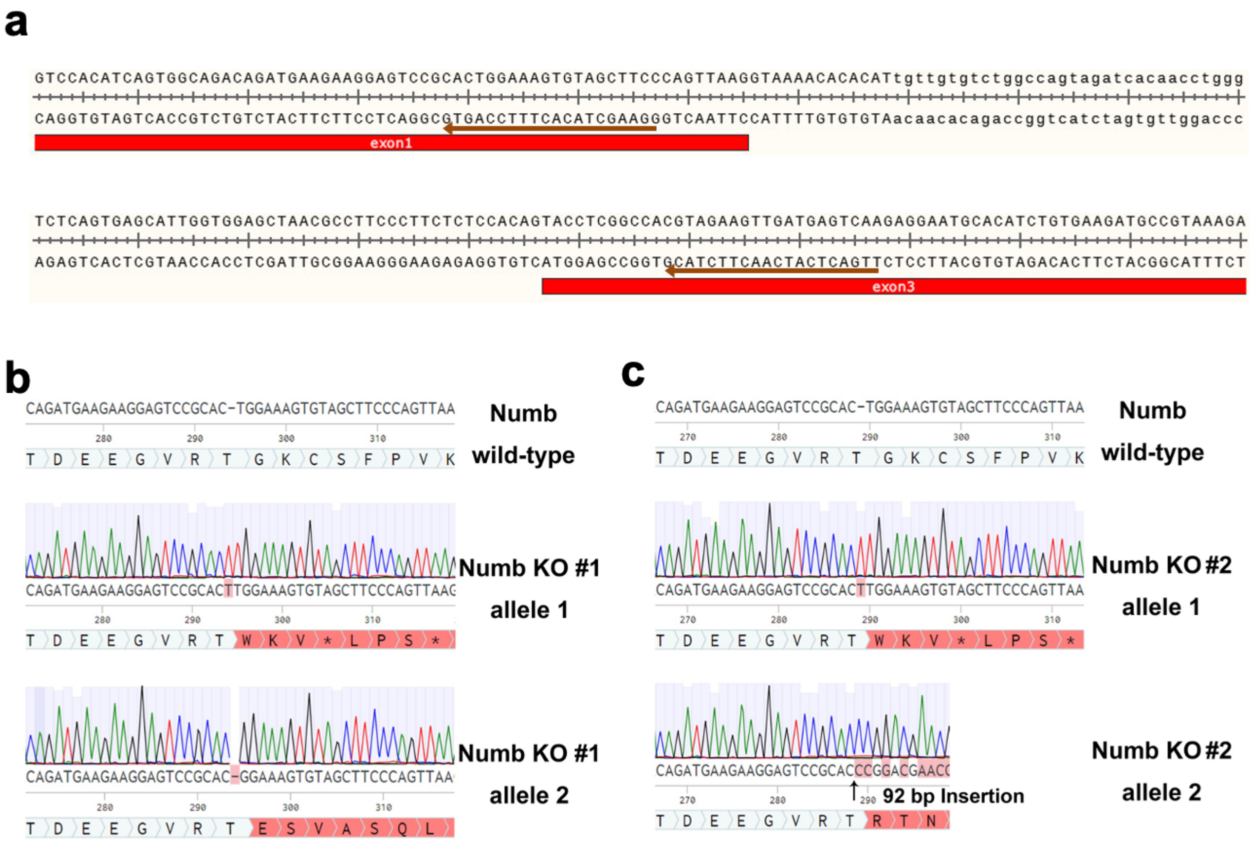

Supplementary Figure 7. Design of *Numb* CRISPR/Cas9 and identification of genomic mutations in individual cell clones.

(**a**) Two guide RNAs were designed to target exon 1 and 3 of mouse *Numb*. Brown arrows indicate the sequence of gRNAs. (**b-c**) The gRNA targeting region in the mouse genome was amplified by genomic PCR, ligated into TOPO vector, and transfected into chemically competent cells. 20 bacterial colonies of each cell clones were randomly picked and sequenced. The sequences are aligned with the *Numb* WT gene sequence from *M. musculus*. In *Numb* KO cell clone #1, a single-base-pair insertion and single-base-pair deletion were present. In *Numb* KO cell clone #2, a single-base-pair insertion and 92bp insertion were present. All mutations cause biallelic frameshift mutations that eventually lead to nonsense-mediated decay (NMD) of the mutant mRNA.

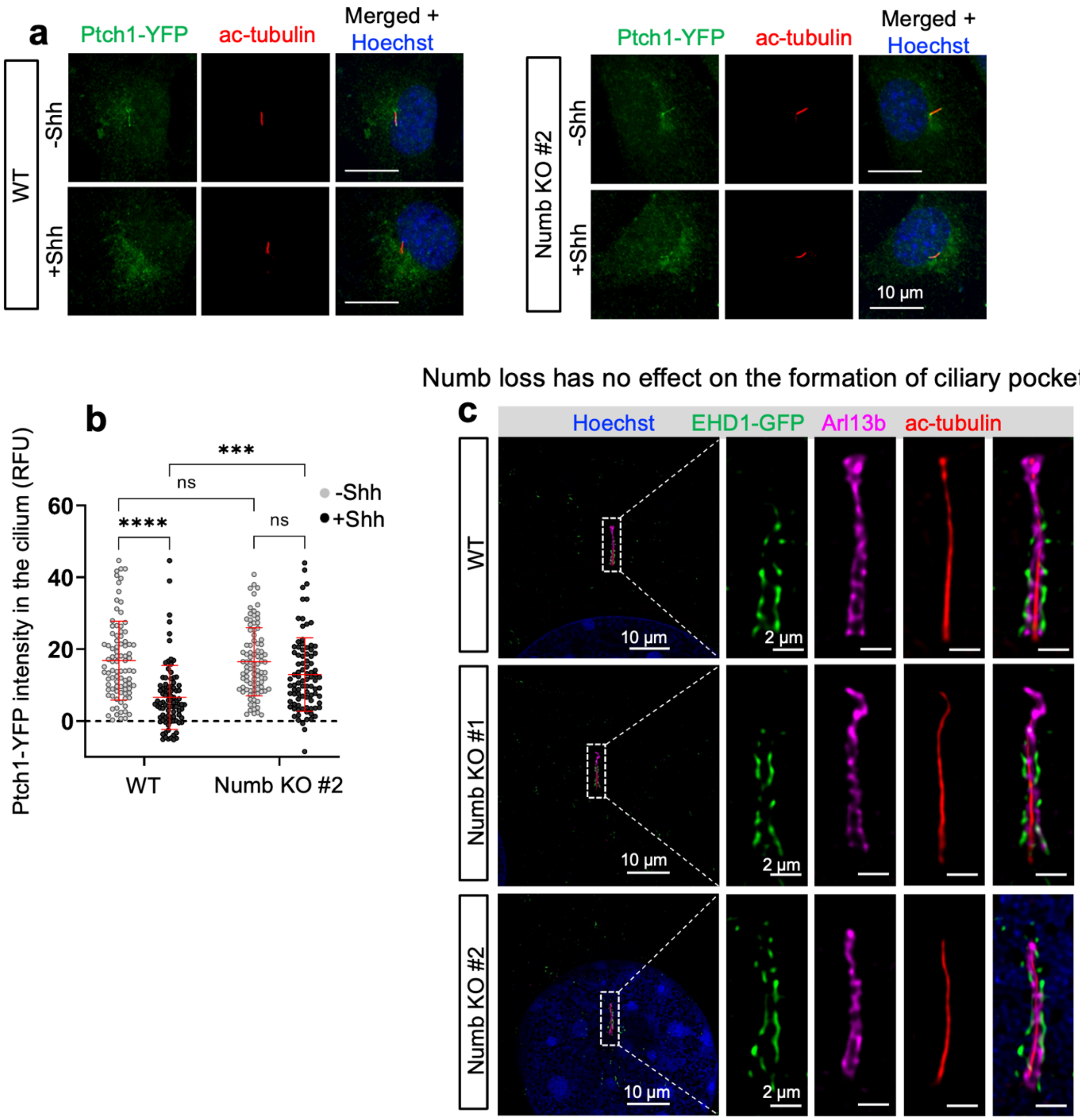

Supplementary Figure 8. Numb is required for Shh-induced Ptch1-YFP exit from the cilium. (**a**) Representative images of Ptch1-YFP in the cilium before and after Shh treatment. NIH3T3 cells were infected with Ptch-YFP lentivirus and treated with Shh or vehicle for 30 min before fixation and staining. Cilia are labeled with acetylated-tubulin (red). (**b**) Quantification of Ptch1-YFP intensity in the cilia. n = 90 cells per condition. Two-way ANOVA with multiple comparisons (Tukey test). Data are shown as mean ± SD. ***, $p < 0.001$; ****, $p < 0.0001$. ns, not significant. (**c**) Numb loss has no effect on the formation of ciliary pocket. Representative images of the morphology of ciliary pocket in WT and *Numb* KO cells. Cells were transfected with EHD1-GFP, underwent expansion microscopy, and imaged with Airyscan microscopy. The image shown is from a single focal plane highlighting the ciliary pocket. The cilium axoneme is labeled with acetylated (ac)-tubulin (red); cilium membrane is labeled with Arl13b (Magenta); ciliary pocket is highlighted by EHD1 (green). Source data are provided as a Source Data file.

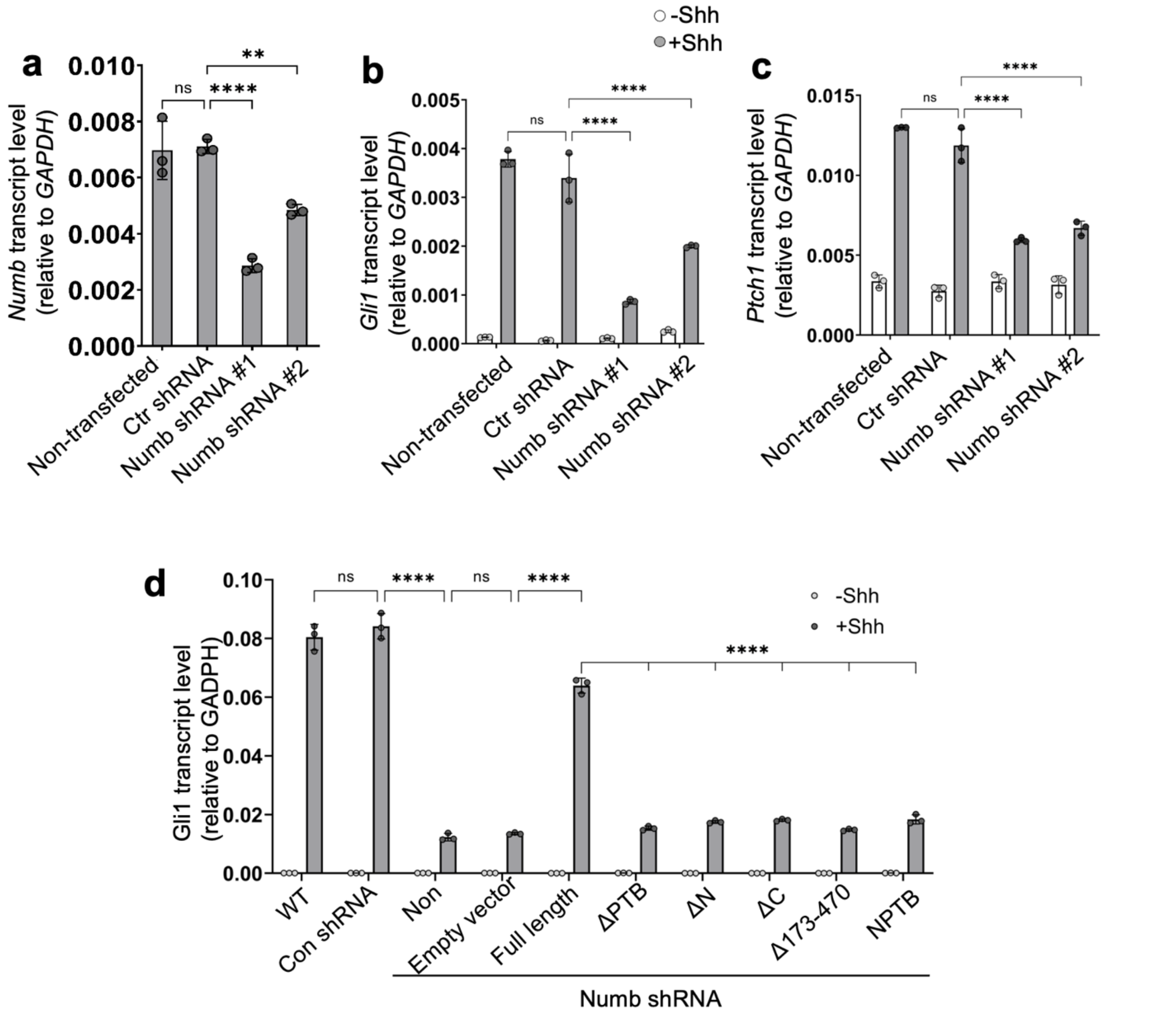

Supplementary Figure 9. *Numb* shRNA impairs activation of Hh signaling.

(**a**) *Numb* shRNA significantly reduced *Numb* gene expression. *Numb* mRNA levels were measured by qPCR. Control (Ctr) shRNA is a non-mammalian targeting shRNA. (**b-c**) 3 days after lentivirus-mediated transfection of *Numb* shRNA, cells were serum-starved for 24 h with or without Shh. Hh signaling activity was evaluated via qPCR measuring transcript levels of *Gli1* and *Ptch1*. *Numb* knockdown reduced Shh-induced Hh target gene transcription. (**d**) The shRNA-resistant Numb and Numb truncated variants were expressed in cells of Numb knockdown. The full-length Numb protein, but not any of the truncated variants rescued Hh signal transduction. Hh signaling was assessed by the levels of *Gli1* transcription.

Statistics in (**a**): One-way ANOVA with multiple comparisons (Tukey test). (**b-d**): Two-way ANOVA with multiple comparisons (Tukey test). Data are shown as mean ± SD. n=3. ****, $p < 0.0001$; ***, $p < 0.001$; **, $p < 0.01$; *, $p < 0.05$. ns, not significant. Source data are provided as a Source Data file.

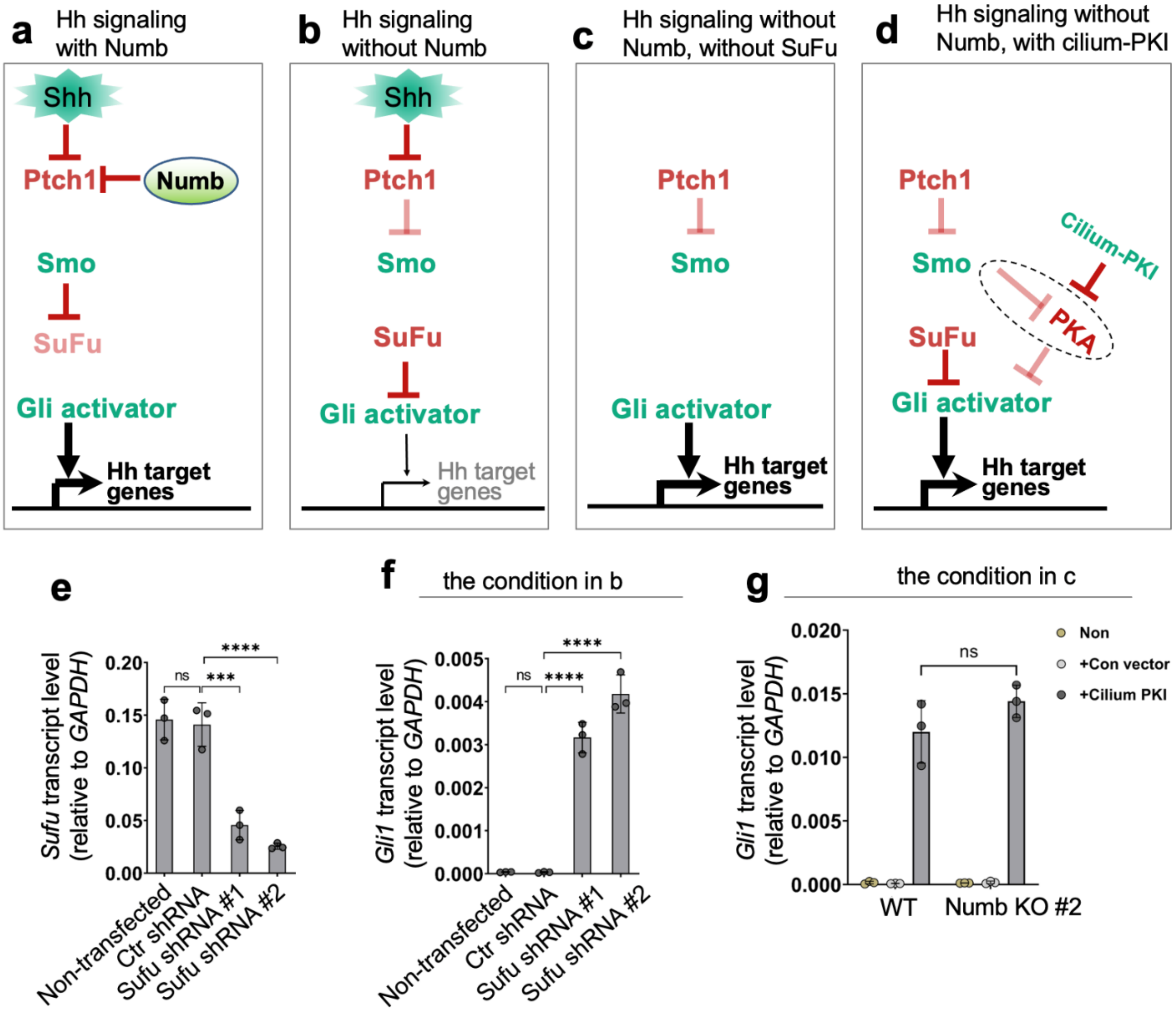

Supplementary Figure 10. Activating signaling components downstream of Ptch1 turns on Hh signaling in *Numb* KO cells.

(**a-d)** Schematic of Hh signaling in various scenarios. (**a**) Numb enhances Hh signaling at the level of Ptch1; (**b**) Numb loss attenuates the Hh signaling activity; (**c**) SuFu knockdown turns on Hh signaling independent of Shh in *Numb* knockout (KO) cells; (**d**) Inhibiting ciliary PKA activity turns on Hh signaling independent of Shh in *Numb* KO cells. (**e**) *Sufu* shRNAs significantly reduced *Sufu* gene expression in *Numb* KO cells. *Sufu* mRNA levels were measured by qPCR. (**f**) In *Numb* KO cells, Hh signaling is turned on by *Sufu* knockdown. Hh signaling activity is assessed by *Gli1* transcript levels. This condition corresponds to the scenario **b**. (**g**) PKI fused with Arl13b-N-RVEP-PR (Cilium-PKI) was expressed in cells via lentiviruses. In *Numb* KO cells, Hh signaling is turned on by cilium-PKI to the levels comparable to those in WT cells. Hh signaling activity is assessed by *Gli1* transcript levels. This condition corresponds to the scenario **c**. Statistics in (**e**, **f**): One-way ANOVA with multiple comparisons (Tukey test). Statistics in (**g**): Two-way ANOVA with multiple comparisons (Tukey test). Data are shown as mean ± SD. n=3 independent experiments. ****, $p < 0.0001$; ***, $p < 0.001$. ns, not significant. Source data are provided as a Source Data file.

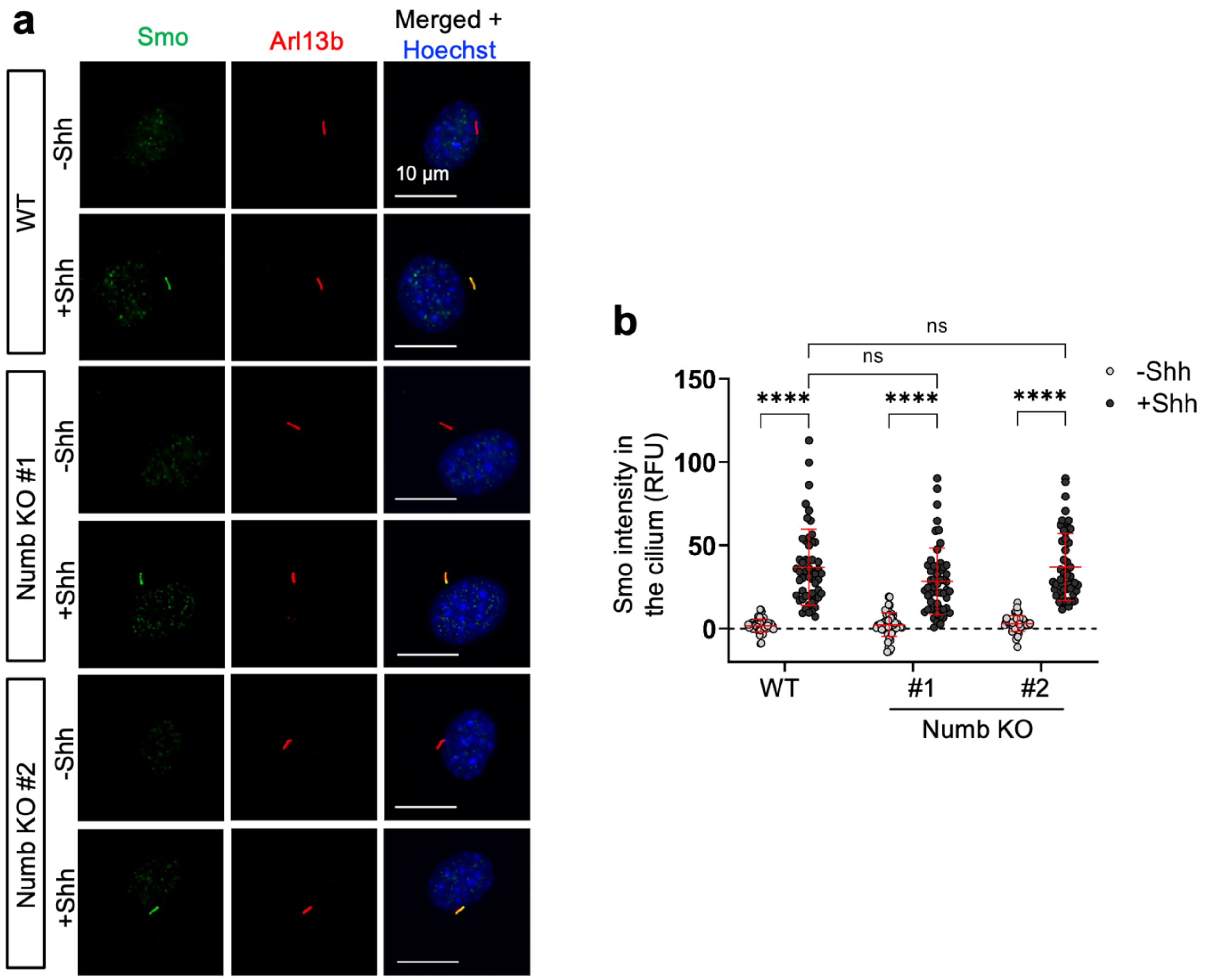

Supplementary Figure 11. The effect of Numb loss on ciliary accumulation of Smo.
(**a**) Immunofluorescence of Smo in the cilium in WT and *Numb* KO cells. Cells were serum-starved with or without Shh in the culture medium for 24 h before staining. (**b**) Quantification of Smo fluorescence intensity in the cilium. Data are shown as mean ± SD. n = 53 cilia were quantified per condition. RFU, relative fluorescence unit. Statistics: Two-way ANOVA with multiple comparisons (Tukey test), ****, $p < 0.0001$. ns, not significant. Source data are provided as a Source Data file.

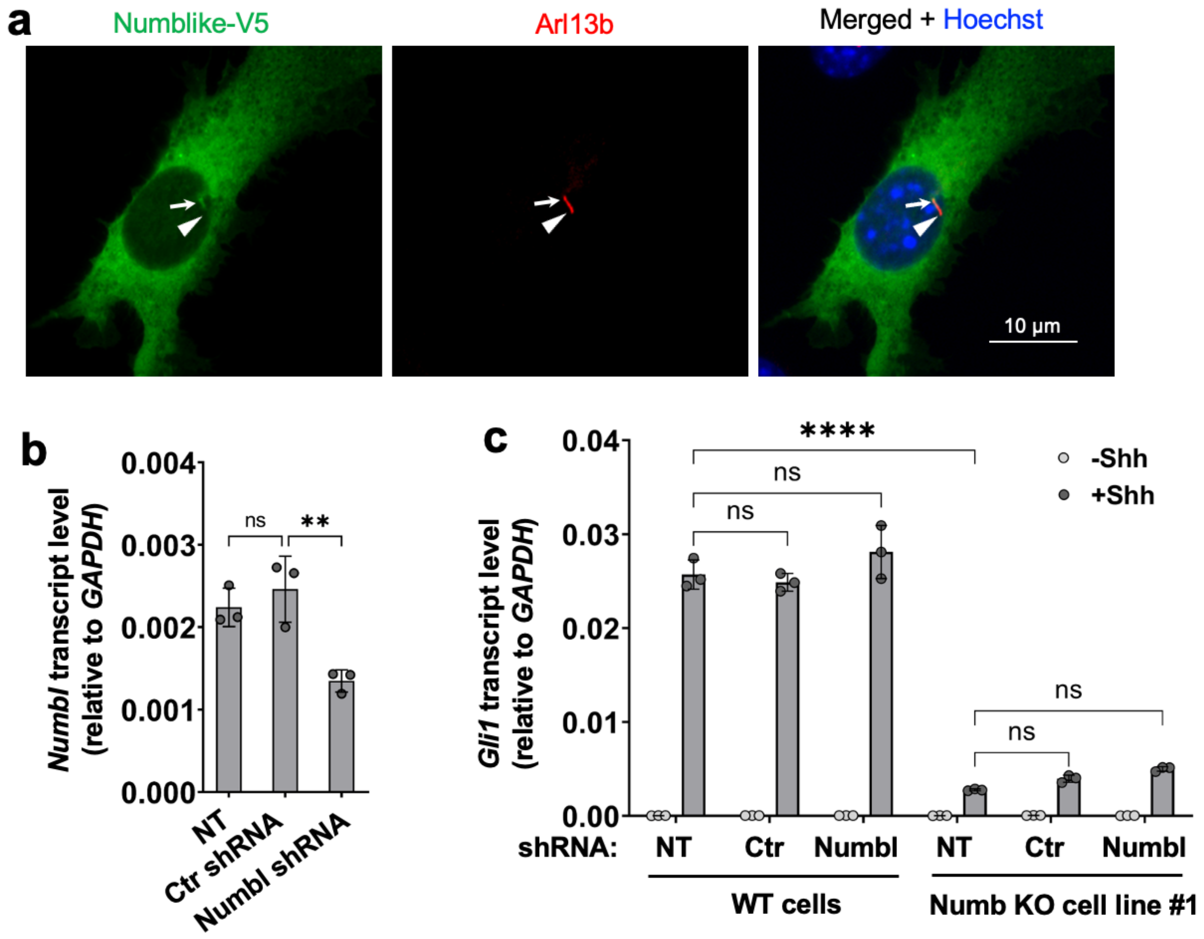

Supplementary Figure 12. Numblike knockdown does not impact Hh signaling.
(**a**) Numblike-V5 was expressed in NIH3T3 cells and co-immunostained with the cilium marker Arl13b. Note that in comparison to Numb (Fig 3a), Numblike diffuses uniformly throughout the cytosol, without displaying punctate localization. (**b**) *Numblike* shRNA significantly reduced *Numblike* gene expression in NIH3T3 cells. *Numblike* mRNA levels were measured by qPCR. (**c**) Parental (WT) NIH3T3 cells or *Numb* KO cells were infected with lentiviruses that express *Numblike* shRNA. 3 days after infection, cells were serum-starved for 24 h with or without Shh. Hh signaling activity was evaluated via qPCR measuring the transcript levels of *Gli1*. Control (Ctr) shRNA is a non-mammalian targeting shRNA. NT: non-transfected.
Data are shown as mean ± SD, n=3. Statistics: one-way ANOVA in (**b**) and Two-way ANOVA with multiple comparisons (Tukey test) in (**c**). ****, $p < 0.0001$; **, $p < 0.01$; ns, not significant. Source data are provided as a Source Data file.

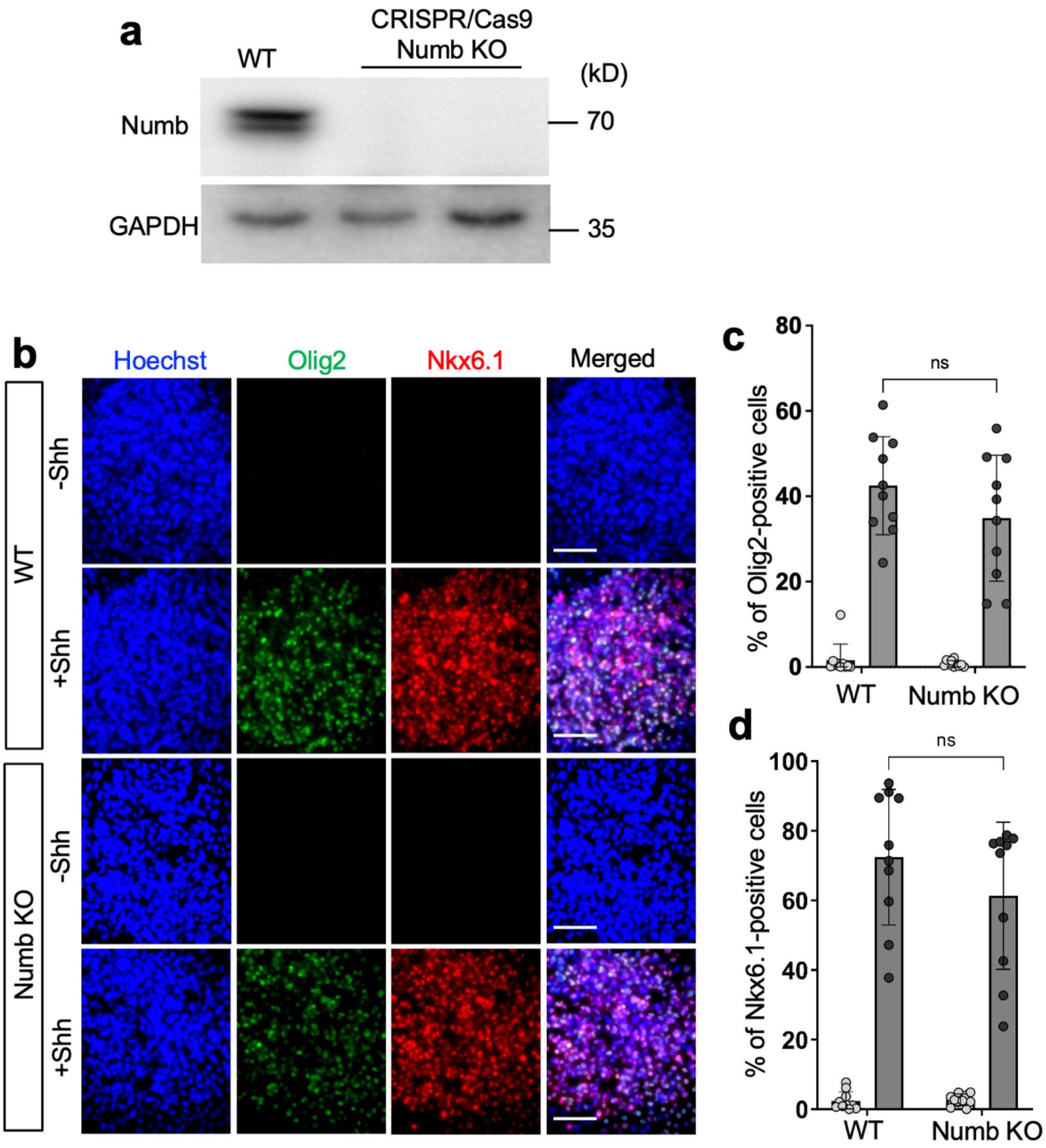

Supplementary Figure 13. Numb loss has moderate or no impact on the differentiation of NPCs that are reliant on medium to low Hh signaling activity.

(**a**) Western blotting results show that Numb protein was ablated in mouse embryonic stem cells. Three independent experiments were performed with similar results. (**b**) Representative images of NPC differentiation into Nkx6.1- and Olig2-positive neural progenitors after 3 days induction. (**c-d**) The percentage of cells positive for Olig2 and Nkx6.1 in WT or *Numb* KO NPCs. n = 10 images from three independent experiments were quantified for each condition. Each image represents one NPC colony consisting of approximately 200–300 cells. Data are shown as mean ± SD. Statistics: Two-way ANOVA with multiple comparisons (Tukey test). ns, not significant. Source data are provided as a Source Data file.

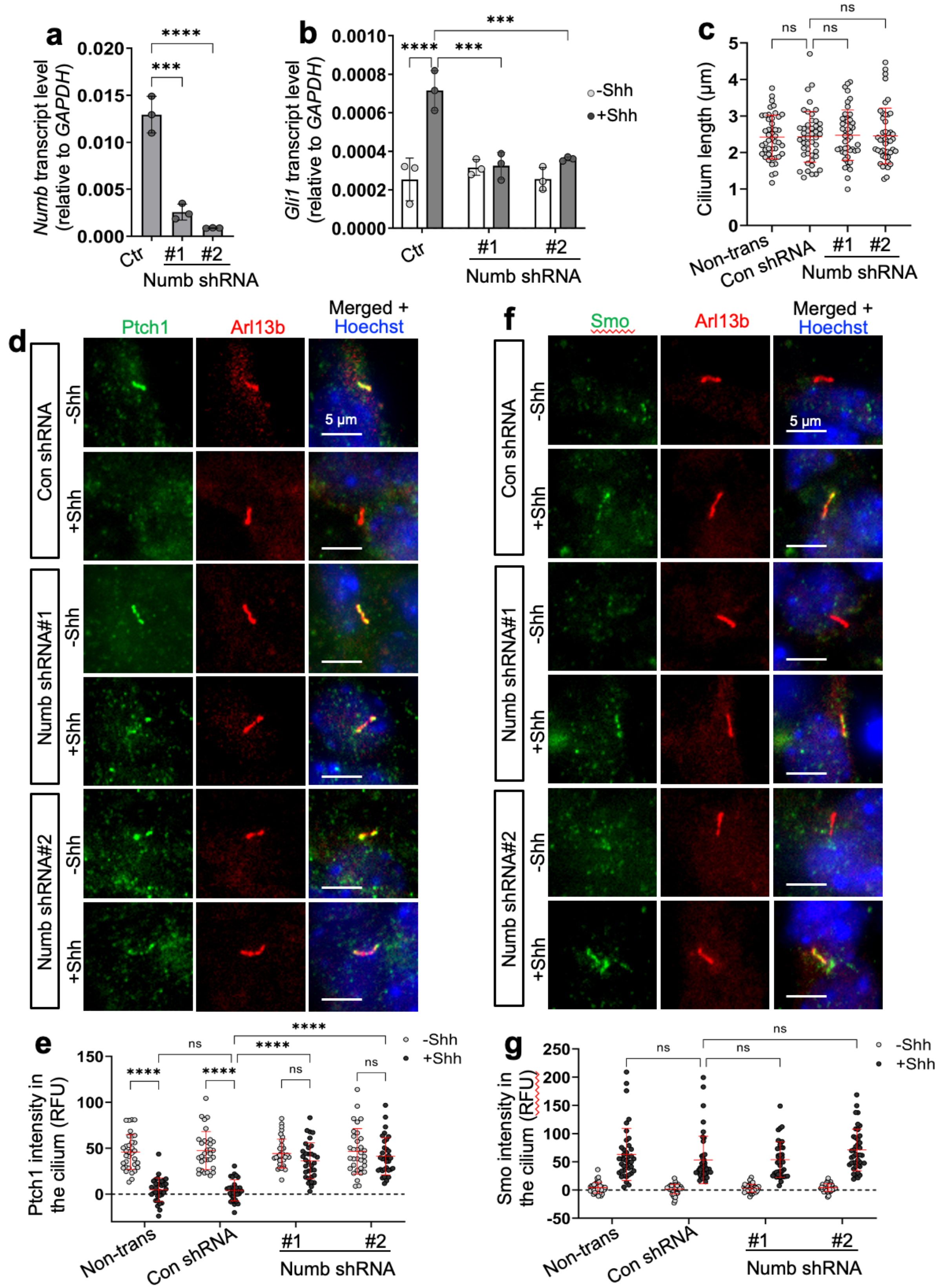

Supplementary Figure 14. Numb is required for activation of Hh signaling and Shh-induced Ptch1 exit from the cilium in GCPs.

(**a**) *Numb* shRNAs significantly reduced *Numb* gene expression in primary cultured GCPs. *Numb* mRNA levels were measured by qPCR. (**b**) *Numb* knockdown reduced Shh-induced Hh target gene transcription. 3 days after lentivirus-mediated transfection, GCPs were stimulated for 24 h with or without 1 μg/ml Shh. (**c**) Quantification of the cilium length in non-transfected, control shRNA or *Numb* shRNA transfected GCPs. 3 days after lentivirus-mediated transfection, GCPs were immuno-stained with Arl13b. n=45 cilia were quantified per condition. (**d**) Immunofluorescence staining of endogenous Ptch1 in the cilia of primary cultured GCPs. 3 days after lentivirus-mediated transfection, GCPs were pretreated with SAG for 24hr to induce Ptch1 expression. After that cells were stimulated with 1 μg/ml Shh or control medium for 1 h before being fixed for immunostaining. Scale bar, 5 μm. (**e**) Quantification of endogenous Ptch1 intensity in the cilia of primary cultured GCPs. n = 32-34 cilia were quantified per condition. (**f**) Immunofluorescence of Smo in the cilia of primary cultured GCPs. 3 days after lentivirus-mediated transfection, GCPs were treated for 24 h with or without 1 μg/ml Shh. Scale bar, 5 μm. (**g**) Quantification of Smo fluorescence intensity in the cilium. N = 41-44 cilia were quantified per condition.

Statistics in (**a, c**): One-way ANOVA with multiple comparisons (Tukey test). (**b**, **e, g**): Two-way ANOVA with multiple comparisons (Tukey test). Data are shown as mean ± SD. ***, $p < 0.001$; ****, $p < 0.0001$. ns, not significant. Source data are provided as a Source Data file.

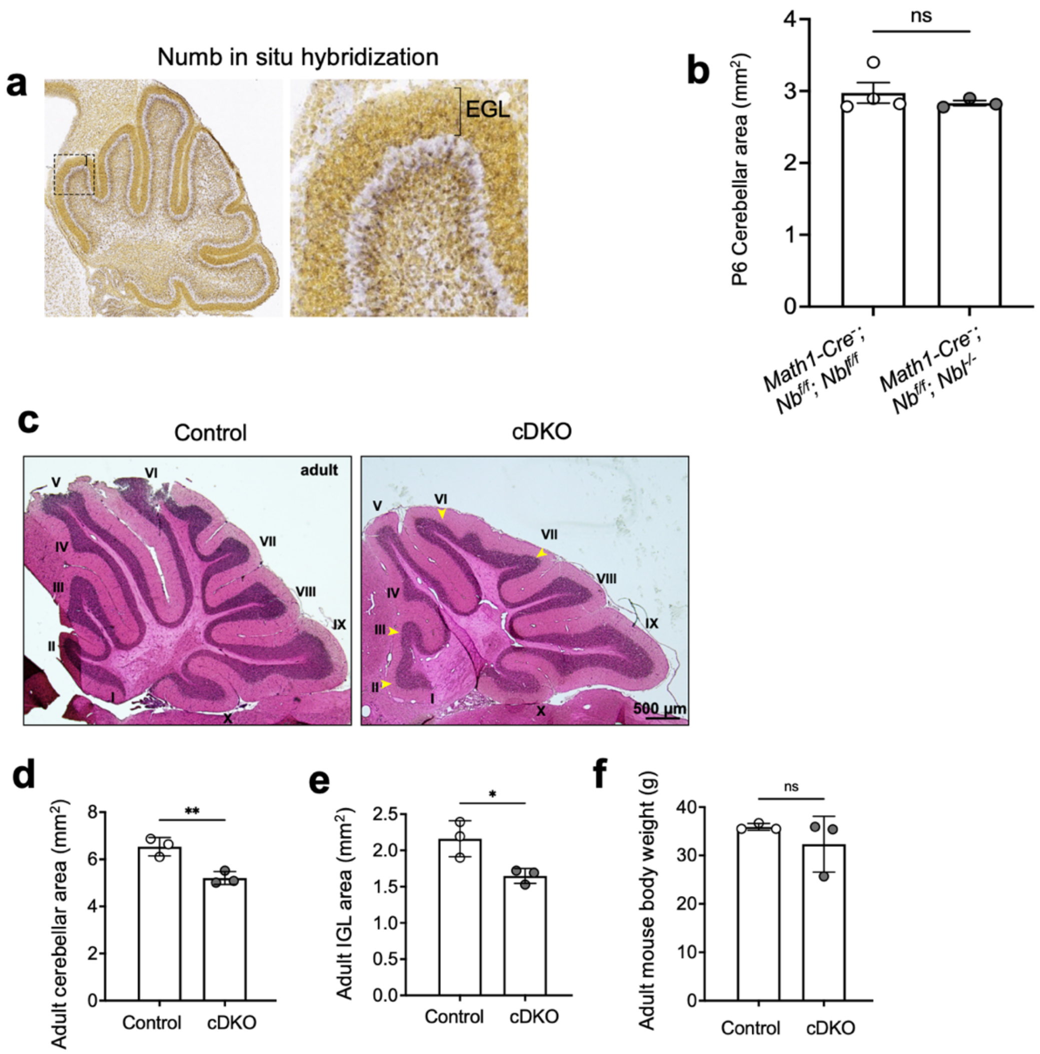

Supplementary Figure 15. *Numb/Numbl* cDKO leads to reduced cerebellar size in adult mice.

(**a**) In situ hybridization on sagittal mouse sections at P4 for *Numb*. *Numb* mRNA is present in the EGL, Purkinje cells, and IGL. Bracket denotes the EGL. Box indicates zoomed area. Images are obtained from The Allen Brain Atlas. (**b**) Quantification of the overall cerebellar area in P6 mice measured at the same mediolateral level. n = 3 cerebella per group. Deletion of *Numbl* has no effect on cerebellar area. (**c**) Sagittal sections of cerebellum from adult control and *Numb;Numbl* cDKO mice with H & E staining. Roman numerals indicate the lobules and arrowheads indicate under-developed lobules in *Numb;Numbl* cDKO cerebella. (**d-e**) Quantification of the overall cerebellar area and IGL area in adult mice measured at the same mediolateral level. n = 3 cerebella per group. **(f)** Body weight in adult mice. n = 3 mice per group.

All results are presented as mean ± SEM. Statistics: Student's t-test, *, p<0.05; **, p<0.01. ns, not significant. Source data are provided as a Source Data file.

Supplementary Methods

Chemicals and other reagents used in this study

**Chemicals**		
Biotin	Sigma	Cat#: B4501
SAG	Sigma	Cat#: 364590-63-6
Purmorphamine	Calbiochem	Cat#: 483367-10-8
Recombinant mouse Shh,N terminus	R&D Systems	Cat#: 461-SH-025
Recombinant human ShhN (C24II) peptide	R&D Systems	Cat#: 1845-SH
Protein A/G Plus-Agarose	Santa Cruz Biotechnology	Cat#: sc-2003
Anti-HA Magnetic Beads	Thermo Fisher Scientific	Cat#: 88836
Streptavidin magnetic beads	Thermo Fisher Scientific	Cat#: 88817
Lipofectamine 2000	Thermo Fisher Scientific	Cat#: 11668019
CalPhos Transfection kit	Thermo Fisher Scientific	Cat#: NC9567834
Mm00494645_m1 (Gli1) Taqman probes	Thermo Fisher Scientific	Cat#: 4331182
Mm00436026_m1 (Ptch1) Taqman probes	Thermo Fisher Scientific	Cat#: 4331182
Mm99999915_g1 (GAPDH) Taqman probes	Thermo Fisher Scientific	Cat#: 4331182
Mm00477927_m1 (Numb) Taqman probes	Thermo Fisher Scientific	Cat#: 4331182
Mm00477931_m1 (Numblike) Taqman probes	Thermo Fisher Scientific	Cat#: 4331182
Mm00489385_m1(Sufu) Taqman probes	Thermo Fisher Scientific	Cat#: 4331182
Retinoic acid	Sigma	Cat#: R2625
**Bacterial Strains**		
TOP10	Thermo Fisher Scientific	Cat#: C404010
Stbl3	Thermo Fisher Scientific	Cat#: C737303
**Recombinant DNA**		
pcDNA3-Arl13b(full length)-eGFP-TurboID	This paper	N/A
pcDNA3-Arl13b(N+C)-eGFP-TurboID	This paper	N/A
pcDNA3-Arl13b(N+RVEP+PR)-eGFP-TurboID	This paper	N/A
pcDNA3-Arl13b(N+PR)-eGFP-TurboID	This paper	N/A
pcDNA3-Arl13b(RVEP+PR)-eGFP-TurboID	This paper	N/A
pcDNA3-Arl13b(PR)-eGFP-TurboID	This paper	N/A
pFUGW-Arl13b(N+RVEP+PR)-GFP-TurboID	This paper	N/A
pFUGW-Arl13b(PR)-GFP-TurboID	This paper	N/A
pGLAP1-EHD1	Christopher J. Westlake Lab, NIH	DOI: 10.1038/ncb3109
pFUGW-Numb-V5	This paper	N/A
pFUGW-Numb-mCherry	This paper	N/A
pFUGW-Numb-GFP	This paper	N/A
pFUGW-Numb-HA	This paper	N/A

pFUGW-Numb-HA(Numb shRNA binding sites mutant)	This paper	N/A
pFUGW-NumbΔPTB-HA(Numb shRNA binding sites mutant)	This paper	N/A
pFUGW-NumbΔN-HA(Numb shRNA binding sites mutant)	This paper	N/A
pFUGW-NumbΔC-HA(Numb shRNA binding sites mutant)	This paper	N/A
pFUGW-Numblike-V5	This paper	N/A
pFUGW-NumbΔPTB-HA	This paper	N/A
pFUGW-NumbNPTB-HA	This paper	N/A
pFUGW-NumbΔN-HA	This paper	N/A
pFUGW-NumbΔC-HA	This paper	N/A
pFUGW-NumbΔ173-470-HA	This paper	N/A
Ptch1-YFP	Matthew Scott lab, Stanford University	DOI:10.1126/science.1139740
pFUGW-Ptch1-GFP	This paper	N/A
pFUGW-Ptch1-V5	This paper	N/A
pFUGW-Ptch1-YFP	This paper	N/A
mCherry-Clathrin	Addgene	Cat#: 55019
peGFP-N1-SMOM2	This paper	N/A
pFUGW-Boc-YFP	This paper	N/A
pFUGW-SMOM2-GFP	This paper	N/A
pFUGW- Arl13b(N+RVEP+PR)-PKI-Flag	This paper	N/A
pLKO.1-puro Non-Mammalian shRNA Control	Sigma	Cat#: SHC002
NUMB MISSION shRNA-1	Sigma	Cat#: TRCN0000105736
NUMB MISSION shRNA-2	Sigma	Cat#: TRCN0000105738
pCAGGS-control shRNA	DOI: 10.1016/j.neuron.2019.04.003	N/A
pCAGGS-Numblike shRNA	DOI: 10.1016/j.neuron.2019.04.003	N/A
Sufu shRNA-1	This paper	N/A
Sufu shRNA-2	This paper	N/A

Quantitative real-time PCR

NIH 3T3 cells were grown in 24-well plates in regular growth medium for 24 h, followed by serum starvation to induce ciliation and addition of SAG or Shh-N conditioned (or control) medium to induce Shh signaling. After 24 h, cells were RNA extracted using Trizol reagent (Invitrogen). Quantitative PCR was performed on 100 ng total RNA per reaction using Quantstudio 3 System (Applied Biosystems) and qPCR reagents (QuantaBio; qScript XLT One-Step RT-qPCR ToughMix, Low ROX). The TaqMan gene expression probes used were Mm00477927_m1 (Numb), Mm00477931_m1 (Numblike), Mm00489385_m1(Sufu), Mm00494645_m1 (Gli1), Mm00436026_m1 (Ptch1) and Mm99999915_g1 and GAPDH to normalize the samples.

For the analysis of the neural progenitor cells (NPCs), RNA extraction, complementary DNA synthesis, and qRT-PCR analysis were conducted as previously described[1, 2]. The following primers were used for qRT-PCR: *Gli1* (F: 5'-ccaagccaactttatgtcaggg-3' and 5'-agcccgcttctttgttaatttga-3'), *Nkx6.1* (F: 5'-cccggagtgatgcagagt-3' and R: 5'-gaacgtgggtctggtgtgtt-3'), Olig2 (F: 5'-agaccgagccaacaccag-3' and R: 5'-aagctctcgaatgatccttcttt-3'), *Nkx2.2* (F: 5'-

cagcctcatccgtctcac-3’ R: 5’-tcacctccatacctttctcc-3’), *Ptch1* (F: 5’-tgacaaagccgactacatgc-3’ and R: 5’-agcgtactcgatgggctct -3’). Transcript levels were calculated relative to *Gapdh* (F: 5’-agtggcaaagtggagatt-3’ and R: 5’-gtggagtcatactggaaca-3’) using the ΔΔCt method.

P6 GCPs were seeded at 2.5×10^6 cells/well in 6-well plates. GCP cells were treated or not with 1 nM recombinant ShhN for 20 h and pellets were directly resuspended in the lysis buffer. RNA was purified using the RNeasy Mini Plus kit (QIAGEN). cDNA was synthesized using the Transcriptor First Strand cDNA synthesis kit (Roche) using total RNA. Real-time PCR mixes were prepared using Perfecta SYBR Green Supermix (Quanta Biosciences). Reactions were performed in triplicate, and the amount of cDNA per reaction was 5 ng. Results were analyzed using the Comparative Ct method. The levels of *Gli1* mRNA were normalized to *Gusb* mRNA. The primers used were *Gli1* F: GCA GTG GGT AAC ATG AGT GTC T and *Gli1* R: AGG CAC TAG AGT TGA GGA ATT GT.

Supplementary References

1. Pusapati, G.V. *et al.* G protein–coupled receptors control the sensitivity of cells to the morphogen Sonic Hedgehog. *Science Signaling* **11**, aao5749-aao5749 (2018).
2. Gouti, M. *et al.* In vitro generation of neuromesodermal progenitors reveals distinct roles for wnt signalling in the specification of spinal cord and paraxial mesoderm identity. *PLoS Biol* **12**, e1001937 (2014).
