# Supplementary material for: Numb positively regulates Hedgehog signaling at the ciliary pocket
Source: Nat Commun. 2024 Apr 25;15:3365. doi: 10.1038/s41467-024-47244-1 (PMC11045789; doi:10.1038/s41467-024-47244-1)
Supplement: Supplementary file 3 — Description of Additional Supplementary Files [file 41467_2024_47244_MOESM3_ESM.pdf]

Description of Additional Supplementary Files

File Name: Supplementary Data 1
Description: Candidates in "Without Shh": the intersection of candidates that meet the criteria in Supplementary Figure 3b and 3c.
Fold change (FC) and adjusted p-value represent Supplementary Figure 3C: (CiliumTurboID: -Shh+biotin)/ (NonCiliumTurboID: +Shh+biotin)

Candidates in "With Shh": the intersection of candidates that meet the criteria in Supplementary Figure 3d and 3e.
Fold change (FC) and adjust p-value represent Supplementary Figure 3e : (CiliumTurboID: +Shh+biotin)/ (NonCiliumTurboID: +Shh+biotin) The P values were adjusted for multiple testing via the Benjamini-Hochberg procedure

File Name: Supplementary Data 2
Description: Proteins in this table are the candidates in the union of the two lists in Supplementary Data 1.
Fold change (FC) and adjusted p-value shown in this table represent Figure 2d: (CiliumTurboID: +Shh+biotin)/ (CiliumTurboID: -Shh+biotin).

The P values were adjusted for multiple testing via the Benjamini-Hochberg procedure.
