# Supplementary material for: Numb positively regulates Hedgehog signaling at the ciliary pocket
Source: Nat Commun. 2024 Apr 25;15:3365. doi: 10.1038/s41467-024-47244-1 (PMC11045789; doi:10.1038/s41467-024-47244-1)
Supplement: Supplementary file 6 — Reporting Summary [file 41467_2024_47244_MOESM6_ESM.pdf]

nature portfolio

Corresponding author(s): Xuecai ge
Frédéric Charron

Last updated by author(s): Mar 12, 2024

Reporting Summary

Nature Portfolio wishes to improve the reproducibility of the work that we publish. This form provides structure for consistency and transparency in reporting. For further information on Nature Portfolio policies, see our Editorial Policies and the Editorial Policy Checklist.

Statistics

For all statistical analyses, confirm that the following items are present in the figure legend, table legend, main text, or Methods section.

n/a	Confirmed	
☐	☒	The exact sample size (*n*) for each experimental group/condition, given as a discrete number and unit of measurement
☐	☒	A statement on whether measurements were taken from distinct samples or whether the same sample was measured repeatedly
☐	☒	The statistical test(s) used AND whether they are one- or two-sided<br>*Only common tests should be described solely by name; describe more complex techniques in the Methods section.*
☒	☐	A description of all covariates tested
☐	☒	A description of any assumptions or corrections, such as tests of normality and adjustment for multiple comparisons
☐	☒	A full description of the statistical parameters including central tendency (e.g. means) or other basic estimates (e.g. regression coefficient) AND variation (e.g. standard deviation) or associated estimates of uncertainty (e.g. confidence intervals)
☐	☒	For null hypothesis testing, the test statistic (e.g. *F*, *t*, *r*) with confidence intervals, effect sizes, degrees of freedom and *P* value noted<br>*Give P values as exact values whenever suitable.*
☒	☐	For Bayesian analysis, information on the choice of priors and Markov chain Monte Carlo settings
☒	☐	For hierarchical and complex designs, identification of the appropriate level for tests and full reporting of outcomes
☒	☐	Estimates of effect sizes (e.g. Cohen's *d*, Pearson's *r*), indicating how they were calculated

Our web collection on statistics for biologists contains articles on many of the points above.

Software and code

Policy information about availability of computer code

Data collection	No commercial or custom code was used to collect data in this study
Data analysis	GraphPad Prism 8 was used for data analysis when ANOVA was needed;<br>For comparison of two experimental groups, Student t-test was performed in Excel or in GraphPad Prism 8;<br>Origin 2019b was used for hierarchical clustering analysis, and volcano plots;<br>GO analysis was done in Metascape;<br>Fiji was used for the measurement of protein intensity in the cilia;<br>Leica LAS X Life Science microscope software was used for the measurement of cilium length.

For manuscripts utilizing custom algorithms or software that are central to the research but not yet described in published literature, software must be made available to editors and reviewers. We strongly encourage code deposition in a community repository (e.g. GitHub). See the Nature Portfolio guidelines for submitting code & software for further information.

Data

Policy information about availability of data

All manuscripts must include a data availability statement. This statement should provide the following information, where applicable:

- Accession codes, unique identifiers, or web links for publicly available datasets
- A description of any restrictions on data availability
- For clinical datasets or third party data, please ensure that the statement adheres to our policy

The original mass spectra have been uploaded to MassIVE (http://massive.ucsd.edu) using the identifier: MSV000090077. The dataset can be downloaded from the MassIVE FTP server with this URL: ftp://massive.ucsd.edu/v04/MSV000090077/. This dataset can also be accessed in ProteomeXchange via Identifier PXD035789. The link is https://proteomecentral.proteomexchange.org/cgi/GetDataset?ID=PXD035789. Mass spectrometry data were processed using Proteome Discoverer v1.5 (Thermo) for quantitative analysis of reporter ion ratios, and the Byonic (Protein Metrics) node to identify peptides and infer proteins against the Mus musculus database from Uniprot.

Research involving human participants, their data, or biological material

Policy information about studies with human participants or human data. See also policy information about sex, gender (identity/presentation), and sexual orientation and race, ethnicity and racism.

Reporting on sex and gender	N/A
Reporting on race, ethnicity, or other socially relevant groupings	N/A
Population characteristics	N/A
Recruitment	N/A
Ethics oversight	N/A

Note that full information on the approval of the study protocol must also be provided in the manuscript.

Field-specific reporting

Please select the one below that is the best fit for your research. If you are not sure, read the appropriate sections before making your selection.

[x] Life sciences [ ] Behavioural & social sciences [ ] Ecological, evolutionary & environmental sciences

For a reference copy of the document with all sections, see nature.com/documents/nr-reporting-summary-flat.pdf

Life sciences study design

All studies must disclose on these points even when the disclosure is negative.

Sample size	No sample size was pre-calculated. Biological and experimental replicates were determined empirically for specific experiments. The sample size was determined based on what is commonly used in the same experiment in literature (see references below) in combination with our own experience and other factors such as the variability of the sample. The sample sizes are included in figure legends.<br><br>• Truong, M.E. et al. Vertebrate cells differentially interpret ciliary and extraciliary cAMP. Cell 184, 2911-2926.e2918 (2021).<br>• Ferent, J. et al. Boc Acts via Numb as a Shh-Dependent Endocytic Platform for Ptch1 Internalization and Shh-Mediated Axon Guidance. Neuron 102, 1157-1171.e1155 (2019).<br>• Klein, A.-L., Zilian, O., Suter, U. & Taylor, V. Murine numb regulates granule cell maturation in the cerebellum. Developmental Biology 266, 161-177 (2004).<br>• Wilson, A. et al. Normal Hemopoiesis and Lymphopoiesis in the Combined Absence of Numb and Numblike. The Journal of Immunology 178, 6746-6751 (2007).<br>• Klein, A.-L., Zilian, O., Suter, U. & Taylor, V. Murine numb regulates granule cell maturation in the cerebellum. Developmental Biology 266, 161-177 (2004).<br>• Pusapati, G.V. et al. G protein–coupled receptors control the sensitivity of cells to the morphogen Sonic Hedgehog. Science Signaling 11, aao5749-aao5749 (2018).
Data exclusions	No data were excluded from the analysis
Replication	All experiments were performed 3 times or more. The proteomic experiment was done once with each condition containing 3 replicates. All results were successfully replicated.
Randomization	For quantification of cultured cells and NPCs, images were taken randomly from the culture dishes, and all cells were quantified in the image.<br>For qPCR experiments, all results were included in the analysis.<br>For animals, after genotyping, all animals of the appropriate genotypes were used for analysis. After sectioning, staining and quality control,

	all cerebellar sections at the same mediolateral levels were used for quantification.
Blinding	For most imaging experiments, images were taken by one person and the quantification were performed by another person who were blind to the experimental conditions.<br>For qPCR experiments and Western blot results, blinding was not possible as experimental conditions were evident from the results.<br>Quantifications were performed using the same pipeline applied equally to all conditions and replicates.

Reporting for specific materials, systems and methods

We require information from authors about some types of materials, experimental systems and methods used in many studies. Here, indicate whether each material, system or method listed is relevant to your study. If you are not sure if a list item applies to your research, read the appropriate section before selecting a response.

Materials & experimental systems

n/a	Involved in the study	
☐	☒	Antibodies
☐	☒	Eukaryotic cell lines
☒	☐	Palaeontology and archaeology
☐	☒	Animals and other organisms
☒	☐	Clinical data
☒	☐	Dual use research of concern
☒	☐	Plants

Methods

n/a	Involved in the study	
☒	☐	ChIP-seq
☒	☐	Flow cytometry
☒	☐	MRI-based neuroimaging

Antibodies

Antibodies used	All antibodies are included in the Key resources table
Validation	All antibodies available commercially contain validation statements on the associated manufacturer's website. In our hands, all antibodies were validated either through Western blot analysis or immunoflurescence to ensure that they matched either the correct band or staining pattern seen in previous publications. For immunofluorescence, the specific antibody was validated by staining with this antibody in cells that expressed GFP-tagged protein of interest.

Eukaryotic cell lines

Policy information about cell lines and Sex and Gender in Research

Cell line source(s)	All cell lines, NIH3T3 and 293T, were purchased from ATCC, the vendor that authenticate all cell lines before distributing to users. MitC-MEFs for mESC culture were purchased form Gibco (Cat #A34959). The mouse embryonic stem cells were a gift from the Briscoe Lab (reference below). The original HM1 mouse embryonic stem cells were purchased from Thermo Fisher Scientific (A34959). All experiments are done in low-passage cell lines to ensure the authenticity of cell lines.<br><br>• Pusapati, G.V. et al. G protein–coupled receptors control the sensitivity of cells to the morphogen Sonic Hedgehog. Science Signaling 11, aao5749-aao5749 (2018).
Authentication	All authentication were performed by the vendor, and only low-passage cell lines were used in experiments to ensure the authenticity of cell lines.
Mycoplasma contamination	All cell lines are mycoplasma negative certified by the vendor ATCC, and routinely monitored in the lab with Hoechst 33258 staining. All other cell lines are mycoplasma negative, tested by ATCC Mycoplasma testing kit (ATCC 30-1012K).
Commonly misidentified lines<br>(See ICLAC register)	None misidentified cell lines were used in this study.

Animals and other research organisms

Policy information about studies involving animals; ARRIVE guidelines recommended for reporting animal research, and Sex and Gender in Research

Laboratory animals	Mouse strain Math1-Cre was purchased from Jackson lab (Strain# 011104).<br>The Numb/Numbl double floxed mouse strain was purchased from Jackson Lab (Strain# 005384, stock name Numb<tm1Zili> Numbl<tm1Zili>/J).<br>Numbl null was obtained from Michel Cayouette (Montreal Clinical Research Institute, Montreal, Canada), and the mouse is described in Bélanger et al. Developmental Cell. 2017: 40, 137-150.<br>Mice were euthanized at the age appropriate for specific experiments: postnatal day 6, or 3 months.

Wild animals	No wild animals were used in these experiments.
Reporting on sex	Mice of both sexes (not determined) were randomly used for experiments.
Field-collected samples	This study did not use field-collected samples.
Ethics oversight	All animal work was performed in accordance with the Canadian Council on Animal Care Guidelines and approved by the IRCM Animal Care Committee.

Note that full information on the approval of the study protocol must also be provided in the manuscript.

Plants

Seed stocks	N/A
Novel plant genotypes	N/A
Authentication	N/A
